# Factors associated with underweight among lactating women in Womberma woreda, Northwest Ethiopia; a cross-sectional study

**DOI:** 10.1186/s40795-017-0165-z

**Published:** 2017-05-26

**Authors:** Sileshi Berihun, Getachew Mullu Kassa, Muluken Teshome

**Affiliations:** College of Health Sciences, Debre Markos University, Debre Markos, Ethiopia, P.O.BOX: 269, Debre Markos, Ethiopia

**Keywords:** Nutritional status, Underweight, Lactating women, WOMBERMA, Ethiopia

## Abstract

**Background:**

Ensuring nutritional status of women is important because the malignant effects of malnutrition are procreated to the next generation through women and their off-springs. Malnutrition causes 3.5 million death of women and children each year and almost 11% of the disease burden in the world. Therefore, this study was conducted to assess nutritional status and factors associated with underweight among lactating women in Womberma woreda, Northwest Ethiopia, 2016.

**Methods:**

A Community-based cross-sectional study was carried out in Womberma woreda, Northwest Ethiopia. A total of 668 lactating women who have 6–24 months of child were included in the study. Study participants were selected using a multistage sampling technique. Data were collected using interview-administered questionnaire. Body mass index (BMI) was used to measure the nutritional status of lactating women. Women’s body weight and height were measured using the standard anthropometric measurement procedures. Data were entered using EpiData software and analysis was done using SPSS software. Descriptive, bivariate and multivariable logistics regression analysis were used to present the findings. Variables with a *p*-value less than 0.05 on multiple variable logistic regression were taken as significant variables.

**Results:**

Lactating women with normal nutritional status (BMI = 18.5–24.99 kg/m^2^) were 498 (74.5%), and underweight women (BMI < 88.5 kg/m^2^) were 170(25.4%). Respondents with less than five family size (AOR: 0.46, 95% CI: 0.26, 0.81, *p*-value = 0.007), women whose age of first pregnancy was less than 18 years old (AOR: 3.72, 95% CI: 2.33, 6.49 at *p*-value = 0.0001), home delivery for the recent child birth (AOR: 2.36, 95% CI: 1.50, 3.72 at *p*-value = 0.0001), and the absence of nutritional education programs in the community (AOR: 5.5, 95% CI: 1.8, 16.79 at *p*-value = 0.003) were the significant variables with underweight of lactating women.

**Conclusions:**

Nutritional status of lactating women in the study area was poor. One fourth of lactating women was underweight. Factors associated with underweight of lactating women include; respondents with less than five family size, women whose age of first pregnancy was less than 18 years old, home delivery for the recent childbirth, and the absence of nutritional education programs in the community. Early childbearing and short birth intervals between births should be discouraged. Programs which encourage institutional delivery and community-based nutritional education are important to improve women nutritional status.

## Background

Nutritional status is considered as one of the major indicators of the overall wellbeing of a population [1, 2]. Malnutrition causes more than 3.5 deaths among women and children every year and accounts 11% of disability adjusted life years globally [3]. The nutritional status of pregnant and lactating women is very important since it also affects the health of their children [1, 2]. Children born from women who became malnourished during pregnancy and lactation are at higher risk of perinatal health problems. So, improving the nutritional status of pregnant and lactating women is crucial for the health of children [4].

Malnutrition is one of the common health problem affecting millions of peoples in developing countries. This contributes to poor health and nutritional status among the population which leads to chronic energy deficiency [5]. The demand for nutrition is higher for lactating women, and it affects the milk composition and production among lactating women, and the health of infants and adulthood life [6, 7]. A study has shown that nutrients like; vitamin A, D, B1, B2, B6, and B12, fatty acids and iodine are important for the optimal level of milk production [6]. Prolonged inadequate caloric intake can also affect the quality and quantity of breast milk production [8]. So, malnutrition in lactating women can induce a metabolic disturbance in the early life of infants, which can result in physiological alteration [7, 9].

Poor nutritional status of lactating women is considered as one of the greatest threat to the world public health and is a serious developmental threat of a country [10, 11]. In Ethiopia, studies have shown a higher prevalence of malnutrition among lactating women [11]. A study conducted in Northwest Ethiopia showed nutritional inadequacy among urban residents [12]. The 2011 Ethiopian demographic and health survey (EDHS) report showed that 27% of women aged 15–49 years were undernourished. Additionally, the report showed that 9% and 6% of reproductive age women were moderately and severely malnourished respectively [4].

There are a limited number of studies conducted to assess the nutritional status of lactating women in Ethiopia. Therefore, this study was conducted to assess the nutritional status and factors associated with underweight among lactating women in Womberma woreda, North West Ethiopia.

## Methods

### Study design, area and period

A community-based cross-sectional study was carried out in Womberma woreda, Amhara region, North West Ethiopia. The woreda is located at 427 km from Addis Ababa, the capital city of Ethiopia and 172 km from Bahir Dar, the capital city of Amhara region. The woreda has a total of 20 districts; 19 rural and one urban. According to the 2015 population statistics of woreda health office, there were 124,177 populations in the woreda. From this, 4822 were lactating women who have 6–24 months of child [13].

### Eligibility criteria

The source population for this study was all lactating women who had 6–24 months of child. Mothers who lived in the area for six or more months were included in the study. Lactating mothers who were seriously ill and physically unable to fit for anthropometric measurement were excluded from the study. Additionally, women who were or who suspect of pregnancy like; those who did not have menstruation after 45 days of childbearing, and women who had doubt for pregnancy were excluded from the study.

### Sample size determination and sampling procedure

The sample size for the study was calculated using single population proportion formula. It was calculated by considering 27% magnitude of underweight from 2011 EDHS report [4], expecting a maximum disparity of 5% between the study, 95% confidence interval (CI), 10% non-response rate and a design effect of two. The final sample size calculated was 668 lactating women.

**Fig. 1:**
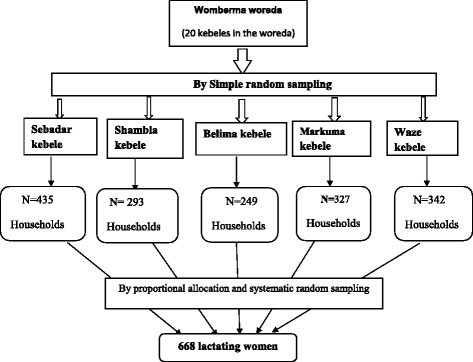
Schematic presentation of the sampling procedure

The study participants were selected using a multistage sampling technique. From the 20 districts in the woreda, five districts were selected randomly by using a lottery method. All households with lactating women were identified from health extension worker’s family folder and proportional allocation was done. Systematic sampling technique was used to select households, and study participants were selected using simple random sampling technique. In households where there are more than one lactating women, lottery method was used to select participants. Three visits to participants were made for absences in the first visit (Fig. 1).

### Data collection techniques and study variables

The data collection tool was developed after reviewing related works of literature. The data collection tool included questions related to socio-demographic variables, obstetric history, nutrition, health-related variables and anthropometric measurements. An adult weighing scale was used for weight measurement, and portable height meter with a moveable head piece was used for the height measurement of the study participants. Data was collected by 12 registered nurses and two supervisors were involved in the data collection process. Training on the data collection procedures and ethical issues were given for the data collectors and supervisors. Body mass index was calculated as weight (kg) divided by height squared (m^2^) for each participant. Instruments were checked against a standard weight for its accuracy daily. Calibration of the indicator against zero reading was checked following weighting every lactating woman. The dependent variable of the study was the nutritional status of lactating women (normal vs underweight). The independent variables include; sociodemographic characteristics, obstetric history, nutrition-related variable, and health-related variables.

### Data quality, processing, and analysis

Data quality was assured through careful design of the questionnaire and data collection procedure. A pre-test was done prior to data collection in the area other than the selected woredas. Data was checked for completeness and consistency. Data was entered using EpiData version 3.1 software and exported to SPSS version 20 for further analysis. Descriptive statistics, binary and multivariable logistic regression analysis were used to present the findings. Variables with a *p*-value less than 0.2 in the binary logistic regression analysis were entered into a multivariable logistic regression analysis to control for potential confounders. Significant association of variables was declared using adjusted odds ratio (AOR) with 95% confidence interval and variables with a *p*-value less than 0.05 were taken as statistically significant.

### Operational definitions

Body mass index: a measurement technique which is calculated as weight in kilogram divided by height in meter squared.

Under-weight: Women whose body weight was too low, BMI < 18.5 kg/m^2^.

Over-weight: Excessive, unbalanced intake of energy or nutritional substances, BMI ≥ 25 kg/m^2^.

Normal weight: Women who have BMI of 18.5–24.99 kg/m^2^.

## Results

### Socio-demographic characteristics of respondents

A total of 668 lactating women were involved with a response rate of 100%. More than half, 382(57.2%) of the respondents were in the age group of 25–34 years and 179(26.8%) were aged greater than 34 years. The mean age and standard deviation (SD) of respondents were 30.4 and 5.4 years respectively. All the respondents were Orthodox Christians by religion and 489 (73.2%) of respondents were unable to read and write. Nearly all, 664(99.4%) participants were farmers (Table 1).

**Table 1:** Socio-demographic characteristics of the respondents in womberma woreda, Northwest Ethiopia, 2016

Variables		Frequency	Percentage
Age (in years)	15-24	107	16
	25-34	382	57.2
	>34	179	26.8
Educational status of the respondent	Cannot read and write	489	73.2
	Can read and write	66	9.9
	Primary (grade 1 to 8)	95	14.2
	Secondary(grade 9 to 10)	18	2.7
Marital status	Married	618	92.5
	Widowed	10	1.5
	Divorced	30	4.5
	Separated	10	1.5
Educational status of husband	Cannot read and write	277	41.5
	Can read and write	175	26.2
	Primary (grade 1 to 8)	129	19.3
	Secondary(grade 9 to 10)	35	5.2
	College level and above	2	0.3
Ethnicity	Amhara	646	96.7
	Agew	16	2.4
	Oromo	6	0.9
Average monthly income (in Ethiopian birr)	<500	356	53.3
	500-999	148	22.2
	1000-1999	39	5.8
	>2000	125	18.7
Family size	≤5	392	58.7
	>5	276	41.3

### Obstetric history of respondents

The mean age of respondents at first marriage was 15 years (SD ± 2.7 years), with a minimum and maximum of 7 and 26 years respectively. The majority, 92.7% of respondents were married before their eighteenth birthday. The mean and SD age of women at first pregnancy was 19.15 ± 2.2 years respectively. The minimum age of pregnancy was 14 years, and the maximum was 28 years. The mean number of children a woman has was 3.7. Only 405(60.6%) of lactating women were using any type of family planning during the time of data collection. Regarding maternal health service utilization, 597(89.4%), 402(60.2%), 163(24.4%) had antenatal visits, institutional delivery, and postnatal visit for their most recent pregnancy and childbirth respectively (Table 2).

**Table 2:** Obstetric history of the respondents in womberma woreda, Northwest Ethiopia, 2016

Variables		Frequency	Percent
Age at first marriage (in years)	<18	619	92.7%
	≥18	49	7.3%
Age at first pregnancy (in years)	<19	452	67.7%
	≥19	216	32.3
Number of pregnancy	≤5	544	81.4%
	>5	124	18.6%
Number of live birth	≤4	513	76.8%
	>4	155	23.2%
Current family planning utilization status	Yes	405	60.6%
	No	263	39.4%
Attending ANC for the current child.	Yes	597	89.4%
	No	71	10.6%
Attending PNC for the current child.	Yes	163	24.4%
	No	505	75.6%
Place of delivery for last pregnancy	Health center	347	51.9%
	Home	266	39.8%
	Health post	16	2.4%
	hospital	39	5.8%
Age of current breastfeeding child	≤12 months	332	49.7%
	>12 months	336	50.3%

### Health and nutritional status of respondents

Respondents were asked if they had any history of sickness in the last 1 month prior to the data collection period. Accordingly, 128(19.2%) reported that they were sick in the last 1 month prior to the data collection. From this, 9(1.3%) of participants had a history of admission related with malnutrition. The average distance of the nearest health facility from respondent’s house was 4.6 km.

The nutritional status of lactating women was measured using BMI. The mean weight (±SD), height (±SD) and BMI (±SD) of respondents were 49.9 ± 5.7 kg, 1.57 ± 0.05 m and 20 ± 1.98 kg/m^2^ respectively. Additionally, 487(72.9%) had a normal nutritional status, and 171(25.6%) were underweight. The magnitude of overweight women was 10(1.5%). Less than half, 259(38.8%) of lactating women received nutritional education in their community, and the main source of such information were health extension workers (Table 3).

**Table 3:** Nutritional and health-related factors for the study participants in womberma woreda, Northwest Ethiopia, 2016

Variables		Frequency	Percentage
Sickness in the last month.	Yes	128	19.2
	No	540	80.8
Admission due to nutrition-related problem in the last one month	Yes	9	1.3
	No	659	98.7
Distance of respondent’s household from the nearby health facility	≤5km	487	72.9
	>5km	181	27.1
Nutritional status (BMI)	Normal nutritional status (18.5-24.99 kg/m^2^)	487	72.9
	Underweight (less than 18.5 kg/m^2^)	170	25.4
	Overweight (> 25 kg/m^2^)	11	1.6

### Factors associated with underweight among lactating women

Variables which were found to be associated with underweight of lactating women on bivariate analysis include; large family size, younger age of the woman at first pregnancy, the absence of male involvement during antenatal visits, place of delivery, and absence of nutritional education programs in the community.

On multivariable logistic regression analysis, variables like; respondents with less than five family size (AOR: 0.46, 95% CI: 0.26, 0.81 at *p*-value = 0.007), women whose age of first pregnancy was less than 18 years old (AOR: 3.72, 95% CI: 2.33, 6.49 at *p*-value = 0.0001), home delivery for the recent childbirth (AOR: 2.36, 95% CI: 1.50, 3.72 at *p*-value = 0.0001), and the absence of nutritional education programs in the community (AOR: 5.5, 95% CI: 1.8, 16.79 at *p*-value = 0.003) were significantly associated with underweight of lactating women (Table 4).

**Table 4:** Multivariable logistic regression analysis showing factors associated with underweight of lactating women in womberma woreda, Northwest Ethiopia, 2016

Variables		Nutritional status of lactating women		COR (95% CI)	AOR (95% CI)	*P*-value
		Normal (BMI 18.5-25 Kg/m^2^) n(%)	Underweight (BMI <18.5 Kg/m^2^) n(%)			
Age	15-34 years old	77 (72.6%)	29 (27.4%)	1	1	
	25-34	290 (76.7%)	88 (23.3%)	1.24 (0.76, 2.02)	1.13(0.59, 2.17)	0.722
	Greater than 34 years old	120 (69%)	54 (31%)	0.84 (0.49, 1.43)	10.81 (0.36, 1.85)	0623
Family size	Less than 5	199 (74%)	70 (26%)	0.99 (0.7, 1.42)	0.46 (0.26, 0.81)*	0.007
	5 and above	288 (74%)	101 (26%)	1	1	
Age at first pregnancy	Less than 18 years old	77 (62.1%)	47 (37.9%)	2.08 (1.38, 3.14)*	3.72(2.13, 6.49)*	0.0001
	18 and above years old	410 (76.9%)	123 (23.1%)	1	1	
Male involvement during ANC follow-up	Yes	189 (77.8%)	54 (22.2%)	1	1	
	No	154 (62.1%)	94 (37.9%)	2.14 (1.44, 3.18)*	0.67 (0.43, 1.04)	0.073
Place of delivery	Home	162 (62.1%)	99 (37.9%)	2.76 (1.93, 3.94)*	2.36 (1.50, 3.72)*	0.0001
	Health facility	325 (82.1%)	71 (17.9%)	1	1	
Attend postnatal visit	Yes	190 (84.8%)	34 (15.2%)	1	1	
	No	297 (68.6%)	136 (31.4%)	2.49 (1.64, 3.76)*	1.25(0.73, 2.12)	0.419
Nutritional education in the community	Yes	219 (86.9%)	33 (13.1%)	1	1	
	No	268 (66.2%)	137 (33.8%)	3.29(2.17, 4.99)*	5.5(1.8, 16.79)*	0.003
Distance from health facility to home	Less than 3 kms	107 (69%)	48 (31%)	1	1	
	3 kms and above	380 (75.7%)	122 (24.3%)	1.39 (0.93, 2.06)	4.53(0.79, 25.72)	0.088

*Statistical significance at *P*-value<0.05, 1: reference group, AOR: adjusted odd ratio; COR: Crude odd ratio

## Discussion

This study was conducted to assess the nutritional status and factors associated with underweight among lactating women in Womberma district, Northwest Ethiopia.

Based on the anthropometric measurement of body mass index, 72.9% of lactating women had normal nutritional status, BMI of 18.5 to 24.99 kg/m^2^ and 25.6% women were underweight, BMI less than 18.5 kg/m^2^. The 2011 EDHS report showed a 27% undernutrition among women [4]. A study conducted in Samre woreda, South Eastern Zone of Tigray also showed a 31% prevalence of underweight [14]. This is higher than the finding of the current study. The reason for the difference between the two studies could be due to the socioeconomic difference, and time difference in which the current study was conducted recently after several community-based interventions were undertaken.

A study conducted in rural Vietnam showed a 23.7% prevalence of malnutrition, which is lower than the current study [15]. The difference could be due to the sociodemographic difference between the two studies. And a similar study conducted among Lactating Women in India also showed a 36.6%, 19.3% and 10% prevalence of underweight among Ladakh, Jammu and Kashmiri women respectively [16]. The difference in the prevalence of underweight between the current study and the study in India could be due to the difference in the sociodemographic and economic differences between the two study areas.

Factors associated with underweight of lactating women were identified. Accordingly, respondents with less than five family size, women whose age of first pregnancy was less than 18 years, home delivery for the recent childbirth, and the absence of nutritional education programs in the community were significantly associated.

Women with less than five family size were less likely to be underweight when compared with women who have higher family size, five and above. This could be because of the food security issue in women with higher family size and related underweight and nutritional depletion of the mother due to successive pregnancies. A systematic review on the effect of births spacing on maternal or child nutritional outcomes showed that short interval between pregnancies is associated with adverse outcome in women’s nutritional status. The review showed significant association of short birth interval with increased risk for maternal anemia and with maternal serum zinc, copper, magnesium, ferritin, folate or thyroid-stimulating hormone [17]. A higher risk of undernutrition among women who have higher family size was also observed in a study conducted in Nekemte, Ethiopia [18].

Women who got pregnant before their eighteenth birthday were 3.7 times more likely to be underweight compared to their counterparts. This could be because of the immature anatomical and physiological conditions in younger women. Poor knowledge of younger women towards dietary intake during pregnancy and lactation could also have an impact on their nutritional status [19]. Studies conducted in Ethiopia also showed that women in the youngest age group (15–19) were more likely to be affected by undernutrition [20, 21]. A similar finding was observed in a study conducted among women of reproductive age in Nepal, which showed that younger women aged 15 to 24 years were almost three times more likely to be malnourished than older women [19].

Respondents who gave birth at home were more than two times more likely to be underweight when compared with women who deliver at a health institution. This could be explained by a reduced risk of obstetric complications in women who deliver at health institutions. Such risks include; hemorrhage which could influence the overall health status of the lactating women. In addition, women who deliver at health facility can get nutrition-related health education from health professionals, which will help them to adapt good behaviors related with nutrition and prevent malnutrition.

Women who did not get a regular nutritional education in their community were more than five times more likely to be underweight than those who got a nutritional education in their local area. This could be due to the positive effect of such educational programs on the healthy behaviors of the community [22]. This finding is similar to a study conducted in Nekemte which showed a lower prevalence of undernutrition in women who got health education about nutrition [18].

The current study has certain limitations. Due to the cross-sectional nature of the study design, it may be difficult to establish a causal relationship between underweight and other independent variables. So, large-scale studies assessing a wide range of socioeconomic factors should be conducted for a better understanding of the factors associated with underweight among lactating women.

## Conclusions

The prevalence of underweight among lactating women was found to be high. Women with large family size, who were pregnant at a younger age, home delivery for the recent childbirth, and the presence or absence of community-based nutritional education programs were significant factors associated with underweight among lactating women. Routine anthropometric measurements should be practiced for pregnant and lactating women for early identification of malnutrition at the community level. Government and other concerned bodies should focus on community-based health education programs on nutritional issues, and prevention mechanisms for early childbearing and short birth interval. Large-scale community-based studies should also be conducted for a better understanding of the nutritional status of lactating women and factors affecting it.

## Abbreviations

ANC: Antenatal care
AOR: Adjusted Odd Ratio
BMI: Body Mass Index
COR: Crude Odd Ratio
EDHS: Ethiopian Demographic and Health Survey
WHO: World Health Organization
